# Automated Verification of IGRT‐based Patient Positioning

**DOI:** 10.1120/jacmp.v16i6.5295

**Published:** 2015-11-08

**Authors:** Xiaojun Jiang, Tim Fox, James S. Cordova, Eduard Schreibmann

**Affiliations:** ^1^ Department of Radiation Oncology and Winship Cancer Institute Emory University Atlanta Georgia; ^2^ Department of Radiology and Imaging Sciences Emory University School of Medicine Atlanta Georgia; ^3^ Medical Scientist Training Program Emory University School of Medicine Atlanta Georgia USA

**Keywords:** verification, quality assurance

## Abstract

A system for automated quality assurance in radiotherapy of a therapist's registration was designed and tested in clinical practice. The approach compliments the clinical software's automated registration in terms of algorithm configuration and performance, and constitutes a practical approach for ensuring safe patient setups. Per our convergence analysis, evolutionary algorithms perform better in finding the global optima of the cost function with discrepancies from a deterministic optimizer seen sporadically.

PACS number(s): 87.55.Qr, 87.55.T, 87.55.N

## I. INTRODUCTION

In radiotherapy, algorithms that implement automated computational checks as software solutions are increasingly used to monitor treatment accuracy and warn clinicians if unusual behavior is found. While software checks have been reported for monitoring treatment parameters,^1^, ^2^ a method for monitoring patient positioning using on‐board imaging has not been implemented. This need arises as for standard fractionation treatments, after acquisition and match by the therapist, the KV and paired DRR images should be reviewed within 24 hrs by the attending physician to confirm the shifts or issue a note if corrections are need to further improve setup in following sessions.

In this study, we report on a software tool that automatically scores registration accuracy to confirm that similar translations and rotations in patient position can be found with an independent system. This assessment does not require human supervision and can therefore be implemented as a software process running in the background for a large number of iterations to achieve exactness in image registration and, subsequently, patient positioning. As an unsupervised system, it can also be used on every fraction and all treatments without requiring additional human resources and effort.

## II. MATERIALS AND METHODS

### A. Registration as a configurable optimization problem

Image registration is essentially the process of matching the patient's anatomy visualized in two images acquired under different conditions. In its manual operation, a user moves one of the images with the mouse to modify the couch translations and rotations until the anatomy is aligned. The automated version is an optimization problem that aims to reproduce the user's actions as a software process where the couch translations and rotation are modified iteratively by a general‐purpose optimizer, each iteration being evaluated to provide feedback on how to adjust the translation/rotations in the next iteration.

The key to automating image registration is developing a mathematical definition of what the user considers perfect anatomical alignment. This is accomplished by the use of a cost function that quantifies in mathematical terms how well images are aligned. A similar dilemma is encountered when selecting the optimization algorithm, with the problem being the presence of local and global minima in the cost function. Minima are solutions that provide the best value for small changes in translations and rotations; however, for larger translations and rotations, there is solution that provides a better match. Various approaches are used by different classes of optimizers when searching for the optimal solution in a trade‐off between speed and accuracy. In the following we will investigate the selection of a robust combination of optimizer and cost function as verified using qualitative testing to check an algorithm's ability for finding the optimal solution. By design, the optimizer and metric were selected to be different from the configuration used in Varian's OBI software (Varian Medical Systems, Palo Alto, CA) that relies on the Mattes mutual information^(3)^ cost function minimized with a gradient descent optimizer.

### B. Cost function selection

To find the best cost function that exhibits the above characteristics, we investigated the following mathematical formulations: normalized cross correlation (NCC), gradient difference, and mean reciprocal squared difference. These formulations are quite popular in the field of image registration for describing the match between two images.^4^, ^5^, ^6^ The first two cost functions use the difference between the voxels in the kV‐ and OBI‐acquired images, having an easy, robust mathematical formulation, but being susceptible to changes in image acquisition parameters such as mAs and kVp that affect image quality. The NCC formulation assumes a direct or linear correlation between the intensity values in the two images, while the gradient difference method takes the gradients of the DRR and OBI images to detect organ borders, and then for every voxel takes their squared differences. By its formulation, the gradient difference method is closer to the therapist's judgments when manually matching images online as the human eye is most sensitive to contrast in images and the gradient measures magnitude of image contrast. Similar to the NCC, the mean reciprocal metric takes the squared voxel intensity difference between the two images, after passing them through a reciprocal function of type $\frac{1}{1-x}$ to enhance the metric's sensitivity to the global minima.

To investigate the characteristics of these search space metrics we plot the cost function value of successive translations of (−30,30) mm for an OBI image retrieved from our clinical database. Ideally these images of the search space would have a single and prominent peak when the images are aligned, and values increasing rapidly when the images are shifted from the ideal values.

### C. Optimizer selection

We investigate the convergence properties of two optimization algorithms: a gradient‐based algorithm and an evolutionary algorithm. The convergence characteristics on 100 cases for both algorithms were tracked to record changes in the metric values during algorithm evolution. The final solution was also compared between the two optimization options, with the evolutionary algorithm serving as gold standard for the gradient‐based as it can escape local minima.

Regular step gradient descent optimization adjusts the transformation parameters so that the optimization follows the gradient of the cost function in the direction of the global minima.^(7)^ In our configuration, we used a minimum and maximum step length of 0.01 and 2, respectively and a relaxation factor of 0.9. The algorithm stops when 200 iterations are reached or when variations in the gradient magnitude are less than 1e−5.

The evolutionary algorithm (one‐plus‐one optimizer) works by perturbing the translation/rotation parameters at each iteration. If the new parameters yield a better result (a lower cost function value), then these become the new solution whose parameters are perturbed more aggressively; otherwise, if the initial estimate yields a better result, it remains the solution of choice and the next perturbation is less aggressive. The settings for this optimizer include a growth factor of 2, and an epsilon parameter selected in our tests at 1.5e−6. The algorithm stops when the maximum number of iterations is reached, set at 2,000 in our tests.

## III. RESULTS

### A. Cost function selection

**Figure 1:**
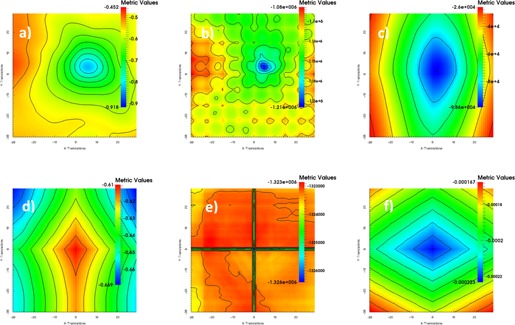
Search space of various cost functions investigated. The display shows match quality under different OBI‐DRR displacements for various mathematical formulations of an ideal match. Ideally, these displays should have a single distinctive blue spot (cost function minimum). The normalized cross correlation (a) displays such a characteristic. The gradient difference metric (b) has the distinct global minima (blue, center) but also has too many "spots," making it unsuitable to be used with a gradient descent optimizer. The mean reciprocal metric (c) has a central spot that is too broad. The lower row shows the same search spaces in the absence of the preprocessing step. These search spaces in the absence of histogram equalization are unusable.

An essential preprocessing step used in our approach was the employment of a histogram matching filter to enhance soft‐tissue visualization in the raw kV images acquired on a Varian OBI system. This is demonstrated in Fig. 1, where typical search spaces for the three types of cost functions investigated are shown with (upper row) and without (lower row) the use of histogram matching as a preprocessing step.

Blue regions represent lower values of the cost function that are associated with the optimal match while red regions represent high values. Ideally, these graphs should have a single blue region representing the ideal value of aligned images and should have smooth color transitions to document consistency or lack of "noise" in the function values when applying small changes. The black lines in the same display represent isocontours of values, similar to the isodose lines used in treatment planning, and are used to illustrate the smoothness of the search space. The NCC cost function shown in Fig. 1(a) displays the most optimization‐friendly behavior, with values descending continuously and monotonously toward the minima independent of the starting position. The gradient difference metric (Fig. 1(b)) has a distinct minimum at the position shown that is desirable in optimization; however, there is a rectangular pattern‐like yellow grid, suggesting the presence of many local minima. With such a search space, the optimization started from different positions will likely result in different solutions as a gradient‐based optimization may become trapped in local minima. The reciprocal metric's search space shown in Fig. 1(c) has a desirable, smooth descent in values, but its minumum is broader when compared to the NCC shown in Fig. 1(a), indicating a less accurate definition of the ideal solution. For comparison, the same metric spaces plotted without the use of a preprocessing filter show that the histogram matching is necessary to create meaningful search spaces for the optimizer. Indeed, in Fig. 1(d) the optima is too broad and at an unlikely location, in Fig. 1(e) the metric space is flat everywhere except in the middle at the known optimal position where its value actually increased, and in Fig. 1(f) the search space is too regular as it does not capture small anatomical details that are obscured by the poor contrast.

### B. Optimizer selection

**Figure 2:**
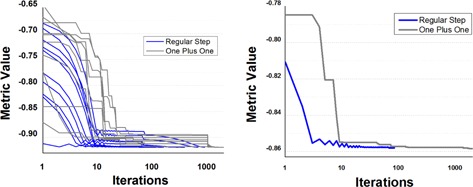
Metric values at each iteration for ten registrations under identical conditions started from various initial positions. A heuristic and a deterministic optimization algorithm are compared. For the case shown in the left panel, all registrations with both algorithms arrived at a similar solution. However for a different case, solutions found by the two algorithms (right) had similar metrics but represented different translations/rotations. See Fig. 4 for details.

The convergence for the two optimization algorithms investigated is shown in Fig. 2, where the cost function values are plotted for ten starts from a different initial value for the one‐plus‐one optimizer (blue lines) and for the gradient optimizer (gray lines). In these optimizations, the NCC metric with the same options is used; therefore, the final metric value should be the similar regardless of the optimizer and the initial position. For the regular gradient algorithm, metric values start large and gradually decrease in various patterns dictated by the initial position but eventually arrive at a similar solution of −0.91888±5.31E−5. A similar behavior is observed for the one‐plus‐one optimizer, but with different patterns. The algorithm starts from a position that is easily improved in the first iterations, hits a plateau at iterations 250–1000 where many random trials do not provide a better solution, and finally arrives at a similar final metric value of −0.91901±1.85E−5. The final metric obtained by this optimizer is slightly better when compared to the gradient optimizer (−0.91901 vs. 0.91888) with a smaller variance (1.85e−5 versus 5.31e−5) indicating better convergence and stability. However, for this case, differences between the two optimizations are small. The final solution is similar for both algorithms, at translations of (−0.0033, −1.3601) mm versus (−0.0047, −1.3066) mm for the regular step and a rotation of $-3.62^{\circ }$ compared to $-3.77^{\circ }$, observed differences being less than 0.1 mm and 0.15°.

**Figure 3:**
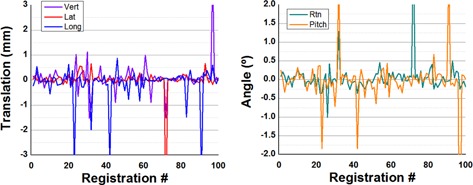
Differences in translations/rotations obtained when comparing the two optimization algorithms. On 7% of the registrations, translational differences were higher than 3 mm, and on 6%, rotational differences were higher than 1°.

However, when repeating the aforementioned test on a set of 100 cases and comparing the final translations/rotations, values were not always consistent. This is shown in Fig. 3, where most of the time solutions found by the compared algorithms match within 1 mm for the translations and within 0.5° for rotations (the mean couch vertical, lateral, and longitudinal differences were −0.15, −0.01, and 0.18 mm, and the mean rotation and pitch angles were 0.02° and 0.04°).

However, occasionally the differences are significant with 5 out of 100 cases having linear discrepancies larger than 2 mm, and 6 cases having angular differences greater than 1.5°. The maximum observed discrepancies for the translations were −10.22, −3.38, and −3.11mm, and $-1.29^{\circ }$ and $-1.01^{\circ }$ for the rotation and pitch angles, respectively.

**Figure 4:**
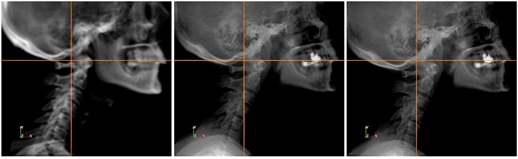
The target DRR image (left), and registered OBI images through the regular step optimizer (middle) and one‐plus‐one optimizer (right) for a case where the two optimizers did not match in final translations and rotations.

Such a clinical case where the optimizers disagreed is illustrated in Fig. 4, where the final solution found by the gradient optimizer (0.183, 0.66, and −0.79mm translations, a couch rotation of $-0.19^{\circ }$ and a pitch of 5.19°) did not concur with the pitch angle from the solution found by the one‐plus‐one optimizer (0.16, 0.66, and −0.40mm translations, a couch rotation of $-0.16^{\circ }$ and a pitch of 2.46°). When visually comparing the two solutions, note that the one‐plus‐one solution has a larger pitch angle to match the high intensity area on the patient's shoulder that is associated with a different position of the skull relative to the shoulders between the DRR and OBI images. Such a case where registrations disagree would need a human observer's review to decide what combination of translation/rotations should be used clinically.

## IV. DISCUSSION & CONCLUSION

Here we present a practical method for quality assurance of the radiation therapist's patient setup, designed to double‐check the couch translations and rotations by repeating the registration with the same clinical images as input, but with an optimizer and cost function differing from the commercial system configuration. In an ideal world, the two systems should provide the same solution independent of the metric and optimizer used. This is not always the case clinically, as image registration accuracy is limited and certain clinical scenarios produce discrepancies that must be investigated in detail.

As documented in the Results section (Fig. 2), our verification algorithm can measure differences from the clinical system as low as a tenth of a millimeter or a degree, adding another layer of confidence that the registration shifts can be properly determined. The convergence analysis in Fig. 2(a) shows that the QA registration will arrive to a similar solution when it is started from shifts of up to 2 cm, being thus able to raise warnings if bulk positioning errors are present.

If the differences between the clinical and QA system are above a user‐defined threshold, an email with a report signaling warnings or errors is sent to the physician. As with any QA system, human (visual) decisions are regarded as the norm, with the physician investigating further on which practical issue caused the discrepancy and on the clinical action to take for each case if a discrepancy is noted by the automated system.
